# Expression of a retinoic acid signature in circulating CD34 cells from coronary artery disease patients

**DOI:** 10.1186/1471-2164-11-388

**Published:** 2010-06-21

**Authors:** Tineke CTM van der Pouw Kraan, Stephan H Schirmer, Joost O Fledderus, Perry D Moerland, Josefien M Baggen, Thomas A Leyen, Anja M van der Laan, Jan J Piek, Niels van Royen, Anton JG Horrevoets

**Affiliations:** 1 Department of Molecular Cell Biology and Immunology, VU University Medical Center, Van der Boechorststraat, 1081BT Amsterdam, The Netherlands; 2 Department of Cardiology, Academic Medical Center, University of Amsterdam, Meibergdreef, 1081BT Amsterdam, The Netherlands; 3 Department of Medical Biochemistry, Academic Medical Center, University of Amsterdam, Meibergdreef, 1081BT Amsterdam, The Netherlands; 4 Department of Clinical Epidemiology, Biostatistics and Bioinformatics, Academic Medical Center, University of Amsterdam, Meibergdreef, 1081BT Amsterdam, The Netherlands; 5 Klinik für Innere Medizin III (Kardiologie, Angiologie und Internistische Intensivmedizin), Universitätsklinikum des Saarlandes, Kirrberger Strasse, 66421 Homburg/Saar, Germany

## Abstract

**Background:**

Circulating CD34+ progenitor cells have the potential to differentiate into a variety of cells, including endothelial cells. Knowledge is still scarce about the transcriptional programs used by CD34+ cells from peripheral blood, and how these are affected in coronary artery disease (CAD) patients.

**Results:**

We performed a whole genome transcriptome analysis of CD34+ cells, CD4+ T cells, CD14+ monocytes, and macrophages from 12 patients with CAD and 11 matched controls. CD34+ cells, compared to other mononuclear cells from the same individuals, showed high levels of KRAB box transcription factors, known to be involved in gene silencing. This correlated with high expression levels in CD34+ cells for the progenitor markers HOXA5 and HOXA9, which are known to control expression of KRAB factor genes. The comparison of expression profiles of CD34+ cells from CAD patients and controls revealed a less naïve phenotype in patients' CD34+ cells, with increased expression of genes from the Mitogen Activated Kinase network and a lowered expression of a panel of histone genes, reaching levels comparable to that in more differentiated circulating cells. Furthermore, we observed a reduced expression of several genes involved in CXCR4-signaling and migration to SDF1/CXCL12.

**Conclusions:**

The altered gene expression profile of CD34+ cells in CAD patients was related to activation/differentiation by a retinoic acid-induced differentiation program. These results suggest that circulating CD34+ cells in CAD patients are programmed by retinoic acid, leading to a reduced capacity to migrate to ischemic tissues.

## Background

CD34+ progenitor cells reside in the bone marrow, and are able to differentiate into mature monocytes, granulocytes, T cells and B cells and endothelial cells. Their multipotential and migratory capacity also enables them to circulate in peripheral blood. Local signals from damaged, ischemic peripheral tissue may provide signals that induce differentiation to cell types that support regeneration of damaged tissue by angiogenesis, myogenesis and neurogenesis. Bone marrow-derived cells expressing CXCR4 are required for neovascularization, secreting factors such as MMP9, and disappear again after oxygen saturation is normalized [1]. Hypoxia induces HIF1a, VEGF and SDF-1, leading to the attraction of hematopoietic precursor cells, which provide a local microenvironment nurturing neovascularisation and neurogenesis [2,3]. Likewise, circulating endothelial precursor cells (EPC) may help tissue repair and angiogenesis by recruiting local mature endothelial cells [4].

In the last decennium, autologous bone marrow-, or peripheral blood derived progenitor cells have been used for intracoronary injection to repair ischemic cardiac tissue after acute myocardial infarction, with contradictory outcomes and overall limited success [5,6]. Aside from technical issues regarding isolation protocols, the functional capacity of autologous stem cells may not be optimal and may differ among patients with coronary artery disease (CAD).

Apart from their regenerative capacities via angiogenesis and myogenesis, progenitor cells are also believed to be involved in the initiation and development of atherosclerotic disease. A low number of peripheral blood CD34+/KDR+ cells is associated with increased cardiovascular events in a group of patients with CAD [7]. Similarly, decreased levels of circulating progenitor cells (CD34+KDR+) were shown to be correlated to increased subclinical atherosclerosis [8]. A recent study has presented evidence of a genetic regulation of EPCs in atherosclerosis [9].

A detailed, genome-wide description of the characteristics of circulating progenitor cells in patients with atherosclerotic disease is currently lacking. This is important knowledge and would be a first step to improve the success rate of autologous progenitor cell transplantation after myocardial infarction. Also, the comparison of the transcriptome of progenitor cells from patients with CAD versus healthy controls might give insights into the role of these cells in the development of atherosclerosis.

In the present study we performed such a genome-wide transcriptome analysis for the first time, presenting a clear signature of circulating progenitor cells. The results indicate that CD34+ cells from patients with CAD have acquired a retinoic acid-induced signature with a low migratory profile, potentially associated with impaired endothelial repair.

## Results

### Patient characteristics and transcriptome analysis of circulating cells

23 patients suspected of significant CAD were referred to the catheterization laboratory. Based on the coronary angiography we selected 12 patients with severe coronary atherosclerosis and 11 well-matched controls without atherosclerotic coronary lesions, further referred to as "controls" [10]. All patients were on aspirin and statin treatment. Baseline characteristics did not differ between atherosclerotic patients and controls except for lipoprotein (a) [10]. The atherosclerotic patient group showed enhanced serum levels of the atherosclerosis marker sICAM, supportive for the vascular inflammatory disease status of these patients, despite medication taken. In CAD patients versus controls, we found no differences in numbers of circulating mononuclear cells, monocytes, lymphocytes, and CD34+ progenitor cells (4.3 ± 1.2 versus 3.7 ± 0.7/μl; p = 0.7), as measured in flow cytometry [10]. The Due to the low circulating numbers, purity of isolated CD34+ cells was generally around 75% in both CAD patients and controls, whereas purity of the other mononuclear cells was >90% [10]. Gene expression analysis for all cell types was performed on Illumina^® ^beadchip microarrays, which proved to be highly reproducible as indicated by very low inter-sample variation among technical replicates (as described before [10]). Despite very low numbers of circulating CD34+ cells we were able to isolate sufficient amounts of RNA from all 23 patients to perform microarray hybridizations. An unsupervised hierarchical clustering of the complete transcriptomes discriminated all different cell types with high specificity, clearly discriminating the highly reproducible transcriptomes of the CD34+ cells from the other mononuclear cells (see additional file 1: Figure S1).

### The transcriptome of CD34+ cells

**Figure 1:**
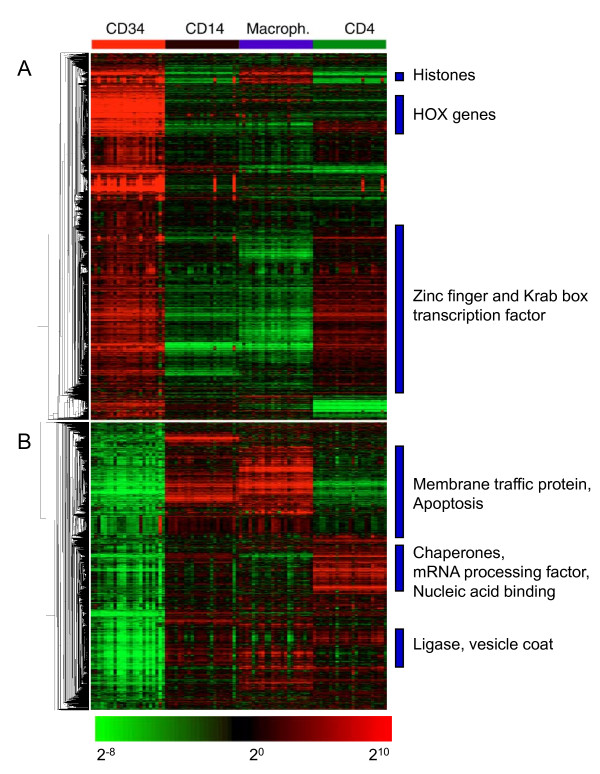
**Differentially expressed genes in CD34+ cells compared to more differentiated cells**. Visualization of expression of 2518 genes that were more highly expressed in CD34+ cells (A), and 1867 genes that were expressed at lower levels (B) compared to each of the three other cell types (indicated at the top). Genes were identified by a pairwise comparison of CD34+ cells with each of the other 3 cell types in 23 individuals. Red indicates a high level of expression, green a low expression level, and black indicates intermediate expression

A paired analysis in Significance Analysis of Microarrays (SAM) [11] was performed on the transcriptome of CD34+ cells compared to the transcriptome of CD4+ cells, CD14+ cells and CD14+ macrophages from the same individuals (n = 23) [10]. At a false significance rate of 5%, we identified 2458 genes that showed high expression in CD34+ cells compared to each of the other cell types, while 1891 genes were expressed at lower levels in the CD34+ cells, visualized by one-way hierarchical clustering in Figure 1. A comparison of our adult CD34+ cell expression profile to that of cord blood-derived CD34+ cells as described by Hemmoranta [12] revealed that the majority of the genes assigned with a HUGO symbol that were expressed in CD34+ cells and not in CD133+ cells, were also expressed in our adult blood CD34+ population (66% n = 71). Although the data cannot be directly compared due to the different platforms used, they suggest a large overlap in expressed genes in CD34+ cells irrespective of age.

Gene ontology analysis using the PANTHER database of protein families was used to classify enriched genes of either higher or of lower expression in CD34+ cells (Figure 1, Table 1 and 2). The "apoptosis signaling pathway" is reduced in CD34+ cells. These genes include anti-apoptotic genes, but predominantly pro-apoptotic genes, suggesting a pro-survival status. A list of the apoptotic pathway genes is provided in additional file 2: Table S1. Analysis of the more highly expressed genes revealed a significant enrichment of genes encoding for nucleic acid binding proteins in CD34+ cells (p = 1.4 × 10^-24^, Table 1), being predominantly of the Zinc finger family with a KRAB domain (129 members were present among the CD34-specific genes). Next, we compared the list of KRAB factor genes to curated genesets in the Molecular Signatures Database (MSigDB) [13], showing substantial overlap with genes that can be induced by the homeodomain containing protein HOXA5 [14]. Furthermore, this set partly overlapped with a series of 10 KRAB factors, identified to be expressed in human CD34+ cells (gene set contributed by Jean-Pierre Bourquin, Dana-Farber Cancer Institute, Boston, USA, unpublished results, Table 3). Significantly, all but 1 of the measurable HOX genes, essential for stem cell self renewal and pluripotency were expressed at significantly higher levels in CD34+ cells with HOXA5 and HOXA9 being most strongly overexpressed (Table 4).

**Table 1:** Gene ontology analysis of genes expressed at higher levels in CD34+ cells

Molecular Function	# genes	p-value
Nucleic acid binding	440	1.41E-24
Transcription factor	328	6.13E-20
Zinc finger transcription factor	173	3.54E-16
KRAB box transcription factor	129	4.95E-16
Synthase and synthetase	63	1.93E-13
Transferase	154	1.13E-11
Synthase	37	1.97E-09
Methyltransferase	34	5.45E-05
Oxidoreductase	93	9.25E-05
Hydrolase	105	5.66E-04
Lyase	32	1.23E-03
Kinase	97	1.43E-03
Transcription cofactor	35	2.58E-03
Synthetase	24	3.41E-03
Other miscellaneous function protein	68	3.98E-03
Nuclease	36	1.19E-02
DNA-directed DNA polymerase	10	1.30E-02
Translation initiation factor	17	1.81E-02
Select regulatory molecule	145	2.39E-02
DNA helicase	19	2.91E-02
Acetyltransferase	20	2.99E-02
Ligase	65	3.82E-02
Aminoacyl-tRNA synthetase	12	3.89E-02

Molecular functions of genes which are upregulated in CD34+ cells compared to more differentiated hematopoietic cells (no significant pathways were identified).

**Table 2:** Gene ontology analysis of genes expressed at lower levels in CD34+ cells

Molecular Function	# genes	P-value
Membrane traffic protein	63	2.40E-13
Nucleic acid binding	280	4.89E-09
Select regulatory molecule	137	4.40E-07
Vesicle coat protein	16	1.53E-05
Chaperone	33	2.31E-05
Other chaperones	21	2.55E-05
Other transcription factor	50	3.29E-04
Ligase	59	6.84E-04
Miscellaneous function	95	7.58E-04
Ubiquitin-protein ligase	36	4.15E-03
Transporter	69	1.61E-02
Phosphatase	34	2.13E-02
Other RNA-binding protein	29	2.35E-02
mRNA processing factor	24	2.80E-02
Transferase	87	3.26E-02
G-protein modulator	49	4.85E-02
Other transporter	42	4.89E-02

**Pathway**	# genes	P-value

Apoptosis signaling pathway	36	3.02E-09
Ubiquitin proteasome pathway	22	1.34E-04
Huntington disease	28	1.10E-02
Ras Pathway	18	2.17E-02
Transcription regulation by bZIP transcription factor	17	3.32E-02
PDGF signaling pathway	28	4.99E-02

Molecular functions and pathways represented by genes expressed at lower levels in CD34+ cells, compared to more differentiated hematopoietic cells.

**Table 3:** KRAB factors enriched in CD34+ cells are regulated by HOXA5

KRAB factor	Human CD34-enriched TF p = 5.31E-07	HOXA5 Targets p = 1.26E-05
ZNF165	+	+
ZNF14	+	+
ZNF228	+	+
ZNF22	+	
ZNF282	+	
ZNF187	+	
ZNF175	+	
ZNF212	+	
ZNF167	+	
ZNF324	+	
ZNF435		+
ZNF136		+
ZNF195		+
ZNF3		+
ZNF20		+
ZNF426		+

Part of the CD34-enriched KRAB domain transcription factors overlap with previously identified transcription factors in CD34+ cells, and HOXA5 target genes. A comparison of CD34-enriched KRAB factors with curated genesets was performed in the MSig database [13]. TF; transcription factors

**Table 4:** Expression of HOX genes is enriched in CD34+ cells.

	CD34	CD14		Macrophages		CD4		average
	avg expr	avg expr	q-value	avg expr	q-value	avg expr	q-value	Fold Change
HOXA5	2117	12	0.0	12	0.0	7	0.0	279.1
HOXA9	1073	30	0.0	18	0.0	10	0.0	76.7
HOXB5	266	19	0.0	18	0.0	18	0.0	16.6
HOXA3	92	7	0.0	7	0.0	7	0.0	15.2
HOXA10	96	12	0.0	11	0.0	10	0.0	9.4
HOXC4	173	23	0.0	24	0.0	37	0.0	7.4
HOXB4	83	14	0.0	12	0.0	55	0.0	5.6
HOXA6	213	77	0.0	63	0.0	82	0.0	3.6
HOXB2	301	131	0.0	202	0.0	752	0.0	1.6
HOXC13	115	186	0.0	204	0.0	137	1.2	-1.3

The average transcript expression level of the individual HOX genes is shown for each cell type. q-values indicate the significance of differential expression compared to CD34+ cells (generated by SAM[11]). The last column indicates the average fold change of the expression of HOX genes in CD34+ cells compared to each of the other cell types, in a paired analysis (23 pairs, and 3 comparisons).

### The CD34+ cell transcriptome of CAD patients indicates a more differentiated phenotype

**Figure 2:**
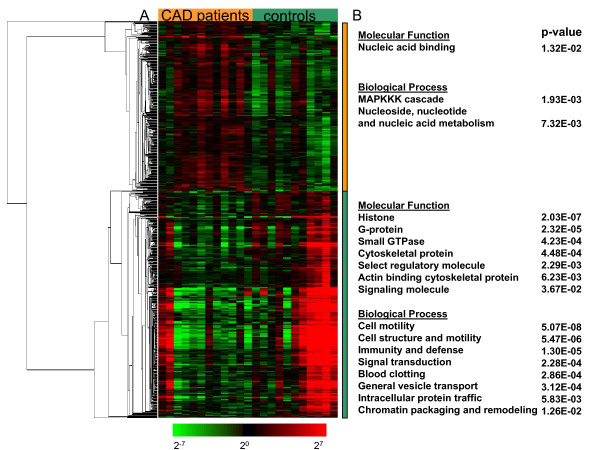
**CD34+ cells from CAD patients show an altered gene expression profile**. Visualization of the expression of 708 genes with significantly different expression levels in CD34+ cells from CAD patients compared to controls.

A two-class statistical analysis in SAM (false discovery rate < 5%) of CD34+ cells identified 303 genes that were expressed at higher levels in CAD patients versus controls, while 405 genes were expressed at lower levels in the CAD patients. A one-way cluster analysis was performed to visualize the correlation of expression of the significant differentially expressed genes, represented in Figure 2A. For a biological interpretation of the differentially expressed genes, we performed a gene ontology analysis in the Panther database and categorized the genes with a specific molecular function or biological process (Figure 2B).

The genes expressed at higher levels in CAD patients display a quite homogenous expression pattern across the different patients and controls, with an average fold change in expression of 1.5. The set of 303 genes which were upregulated in CAD patients was enriched for genes with nucleic acid binding properties and 11 genes involved in the "MAPKKK cascade", including MAP3K12/MUK, MAPK11/p38 beta, MAPK12/p38-gamma, and MAPK7/ERK5 and 60 genes involved in "Nucleoside, nucleotide and nucleic acid metabolism", including transcription factors, DNA- and RNA helicases, RNA polymerases and mRNA splicing factors, suggesting a higher transcriptional activity.

The genes expressed at lower levels in CAD patients display more interindividual variation between patients and controls with an average fold difference in gene expression of -2.4.

**Figure 3:**
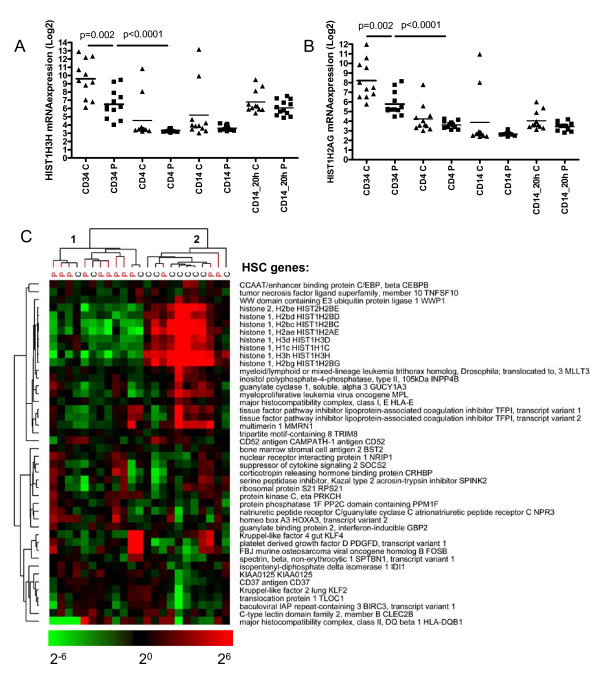
**CD34+ cells from CAD patients are less primitive**. Histone gene expression is decreased in CD34+ cells from CAD patients. mRNA expression levels are shown of two representative histone genes, HIST1H3H (A) and HIST1H2AG (B) in all studied cell types in CAD patients (squares) and controls (triangles). C: Several histone genes belong to the more "primitive" HSC genes [15] and are expressed at lower levels in CD34+ cells from CAD patients.

A set of 19 genes were enriched in the "histone" category. Of these 19 histone genes that were expressed at lower levels in CD34+ cells from CAD patients, 13 were enriched in CD34+ cells compared to more differentiated cells (Figure 1). We included 2 representative examples in Figure 3A and 3B. A more detailed analysis showed that 8 of the 19 histone genes that were downregulated in CAD patients' CD34+ cells in our study belonged to the most undifferentiated hematopoietic stem cells (HSC) transcriptome as identified by Georgantas et al [15]. This HSC-specific gene set was generated from a comparison of the transcriptomes of CD34+/CD38-/Lin- (HSC-enriched) *versus *CD34+/[CD38/Lin]++ cells (HPC-enriched, HSC-depleted) from normal human adult donor bone marrow, neonatal placental/umbilical cord blood, and mobilized adult donor peripheral blood stem-progenitor cells [15].

An unsupervised cluster analysis of the HSC-specific genes from our CD34+ cells divided most patients and controls in separate branches (Figure 3C, Fisher exact, p = 0.04), indicating that CD34+ cells from CAD patients showed a more differentiated profile than CD34+ cells from controls. Because EPCs were earlier shown to correlate with cardiovascular end points, markers of the endothelial lineage (von Willebrand factor and CD133/PROM1) were tested in the CD34+ progenitor cells in this study. Although both markers were expressed at significantly higher levels in the CD34^+ ^versus other mononuclear cell types, their levels did not vary between our control and CAD patient groups.

### The gene expression profile of CD34+ cells from CAD patients correlates with a RA-induced profile

**Figure 4:**
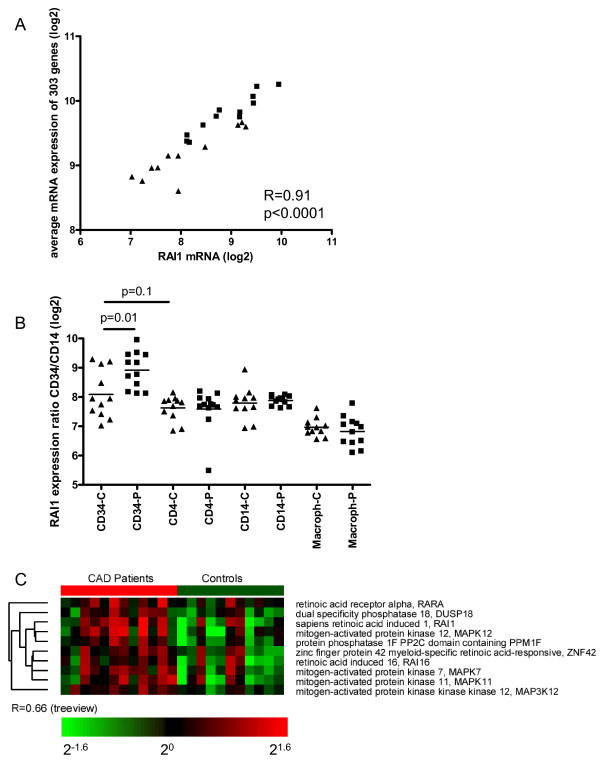
**A retinoic acid-induced signature in CD34+ cells from CAD patients**. A. The induced signature in CD34+ cells from CAD patients correlates with the expression of RAI1. The average expression of the 303 genes of the induced signature of CAD patients is plotted against the expression of RAI1 (CAD patients are represented by squares, controls by triangles). B. Retinoic acid-induced 1 gene expression in different cell types in CAD patients and controls. C. The expression of RAI1 is shown for all studied cell types in CAD patients (squares) and controls (triangles).

Because the 303 genes with a higher expression in CAD patients showed a highly correlated profile, suggesting a common inducer of this profile, we set out to analyze whether we could find genes/transcription factors that could be causal for this profile by searching for the genes that best represent this cluster. For this purpose we calculated the average expression of the 303 genes in each individual patient and control, and subsequently identified genes from the complete data set of all 8943 expressed genes in CD34+ cells that correlated best with this average profile. Interestingly, among the top 12 best correlating genes with this profile in CD34+ cells was the transcriptional regulator RAI1, (Retinoic acid-induced 1, R = 0.91, Figure 4A). RAI1 was also expressed at significantly higher levels in CAD patients (FDR = 4.3%), and indeed is located in the middle of the 303 gene cluster in Figure 2. The other genes which showed a good correlation with the average profile of 303 genes could not be tracked back to a specific transcription factor or other factor which may be causal for increased gene expression. Because Retinoic acid (RA) is a well known modulator of stem cell differentiation, we next focused on genes that are involved in RA-signaling. We identified three RA-related genes that showed a highly correlated expression with RAI1, but did not reach statistical significance in the SAM list of 303 genes at an FDR of 5%. Two of them are known to be induced by RA; RAI16 (retinoic acid induced 16, R = 0.84, p < 0.0001), and ZNF42 (zinc finger protein 42, myeloid-specific retinoic acid-responsive, R = 0.80 p < 0.0001) and its receptor RARα2 (retinoic acid receptor, alpha, transcript variant 2, R = 0.68, p = 0.0003). The combined expression values of all 4 genes also showed a high correlation coefficient (R = 0.91, p < 0.0001) with the average expression of the 303 genes expressed at higher levels in CAD patients (R = 0.91, p < 0.0001, data not shown), which is suggestive for a causal role of RARα stimulation by its ligand, all-trans retinoic acid, in the upregulation of genes in CD34+ cells in CAD patients.

To confirm that the analysis of genes which correlate with RAI1 is related to retinoic acid exposure, we also subjected all genes which showed a positive correlation with RAI1 (R > 0.60, 1304 genes, including 75% of the 303 significant genes) to a pathway analysis in Metacore™. In this case the most significant pathway was "Ligand-Dependent Transcription of Retinoid-Target genes" (p = 2 × 10^-4^), indicating that RAI1 is a good marker for RA-induced transcriptional change. Next, we compared the expression of RAI1 in CD34+ cells from CAD patients and controls to the different peripheral blood cell types, depicted in Figure 4B. Increased expression of RAI1 is restricted to CD34+ cells from CAD patients, suggesting an actively induced expression.

### The CD34+ cell transcriptome of CAD patients indicates a compromised migratory capacity

**Figure 5:**
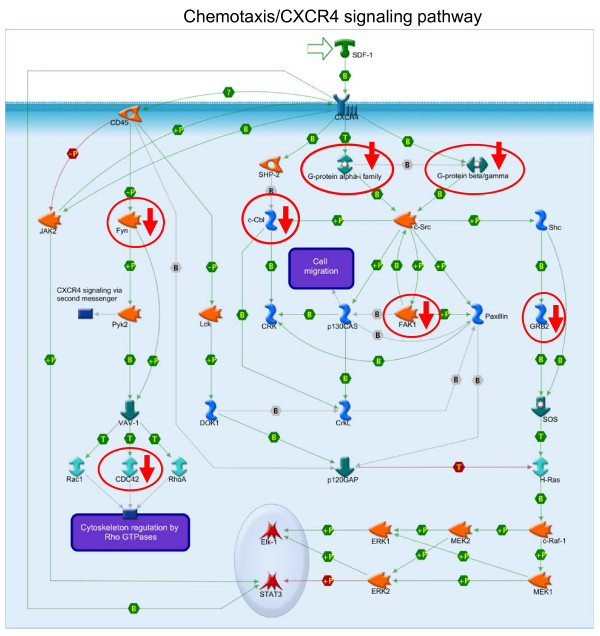
**The CXCR4 pathway is affected in CD34+ cells from CAD patients**. Genes with a lower expression in CD34+ cells from CAD patients represent the Chemotaxis CXCR4 signaling pathway (P = 2.1 × 10^-3^). Genes expressed at significantly lower levels are indicated by a red arrow and are encircled for clarity.

Next to the gene ontology analysis, we also performed an analysis in Metacore™ systems biology suite, which revealed a reduced migratory capacity in CD34+ cells from CAD patients (additional file 3: Figure S2). To search for a possible relation to RA, we performed a pathway mapping analysis in Metacore™ on genes which were negatively correlated with RAI1 (R < -0.60, 460 genes). The most significant pathway, represented by these genes was "Chemotaxis/CXCR4 signaling pathway" (p = 2 × 10^-6^). These results suggest that RA may indeed have downregulated the genes involved in adhesion and migration. CXCR4 signal transduction depends on the activity of small GTPases [16]. We identified a reduced expression of this protein family, including guanine nucleotide binding protein (G protein), alpha z polypeptide (GNAZ, Fold Change, FC = -5.41), GNB5, FC = -4.22, GNG11, FC = -4.36, and GNG8, FC = -11.36. The categories "cytoskeleton protein", and "cell structure and motility" represented by genes with low transcript levels, indicate that the more downstream effector genes of CXCR4 signaling were also expressed at lower levels. These include cell division cycle 42 (CDC42), a small GTPase of the Rho-subfamily (FC = -2.1) (Figure 5), important for migration and adhesion, and other genes in the Rho/cytoskeleton pathway: myosin light chain (MYL) 9 (FC = -11.7), MYL6 (FC = -1.4) and myosin light chain kinase, MYLK (FC = -4.9). (see additional file 4: Figure S3). Migratory and adhesive function may also be affected by the lower expression of the integrins: fibronectin receptor beta/integrin beta 1 (ITGB1, FC = -3.2) and ITGB5 (FC = -6.6), and the tetraspanins CD9 (FC = -2.3) and CD151 (FC = -4.4). (see additional file 3: Figure S2).

## Discussion

In this study, we generated a CD34+ cell-enriched transcriptome from a pair-wise comparison of the gene expression profiles from peripheral blood CD34+ cells with those from other common differentiated hematopoietic mononuclear cells from the same individual. Almost all previous profiling studies on CD34+ cells were focused on bone marrow-derived cells, or G-CSF-mobilized CD34+ cells from peripheral blood. A paired transcriptome analysis of adult CD34+ cells with other blood cell types has not been performed before, and allowed us to identify the transcriptome of CD34+ cells without the influence of interindividual genetic and environmental differences. In addition, a comparison of the CD34+ cell transcriptome from CAD patients with that of controls identified differentiation characteristics and reduced migratory/adhesive capacity of CD34+ cells from the patient group, related to a retinoic acid-induced program.

To the best of our knowledge, this is the first report on the highly significant increased expression of the zinc finger KRAB domain-containing transcription factors in circulating CD34+ cells (129 in total). This relatively novel family of transcription factors is thought to mediate suppression of transcription. The specific function of many KRAB factors is still unknown, but inhibition of RNA polymerase I, II, and III promoters and RNA splicing has been reviewed [17]. Recently, it has been suggested that the repressor complex, containing KAP1, may autoregulate the expression of the KRAB-ZNF family [18]. A model has been proposed in which the zinc finger domains determine the specificity of DNA binding, while the KRAB domain binds to KAP1 (KRAB-associated protein 1), which recruits chromatin remodeling proteins that cause the formation of heterochromatin (condensed inactive DNA) and thus gene silencing [17]. The expression of KAP1 was also significantly higher in CD34+ cells than in the other cell types (data not shown). Thus, our data indicate a specific molecular signaling pathway by which CD34+ precursor cells are kept in a quiescent state compared to other peripheral blood mononuclear cells. From the comparison of the CD34+ gene set with curated gene sets in the MSig database [13], it became clear that there was a significant overlap of several KRAB factors expressed in CD34+ cells and in cells with HOX (homeobox) A5 overexpression [14]. Indeed we identified a high expression of HOXA5 transcripts in CD34+ precursor cells, which could therefore explain the expression of a subset of the KRAB factors. Based on their similarity in protein structure, the eight other HOX genes that were enriched in CD34+ cells may possibly regulate the expression of other subsets of KRAB-ZNF transcription factors. There are 39 members of the HOX gene family in the mammalian genome. These homeobox genes encode for proteins with a helix-turn-helix motif (the homeo domain), which binds to specific sequences of DNA, allowing them to function as transcription factors, important during embryonic development, but also throughout life in lineage-specific cell differentiation from precursor cells [19]. Most of their functions have been assigned through over- or under expression and include both self-renewal, and differentiation towards several hematopoietic cell types [19]. The expression of HOXA9 is particularly important for differentiation into endothelial precursor cells (EPC) [20]. In this study, the expression of all but one of the measurable HOX genes was CD34-enriched, consistent with their differentiation potential.

We compared the transcriptome of CD34+ cells from CAD patients to accurately matched controls, with identical medication (a comparison of the other cell types is reported elsewhere [10]). Although the number of circulating CD34+ cells between CAD patients and controls did not differ, consistent with the previously reported lack of association of CD34+ cell counts with carotid intima-media thickness [8], the transcriptome did show marked differences. We identified lower transcript levels of histone encoding genes in CD34+ cells from CAD patients. This is remarkable because histone encoding genes are typically expressed in early HSC, suggesting a shift towards a more differentiated phenotype of CD34+ cells in CAD patients [15]. These results would imply that CD34+ cells in CAD patients have been pre-activated in-vivo. Corresponding with an activated phenotype, we observed a higher expression of several members of the MAPkinase family in CAD CD34+ cells. The role of an activated MAP kinase pathway in CAD patients is hitherto unknown. MAP kinases are regulated by a broad range of environmental stimuli, such as growth factors and stress (reviewed in [21]). A downstream target of all of the upregulated MAP kinases (ERK5 (MAPK7), p38β (MAPK11), and p38γ (MAPK12)), is the transcription factor Myocyte Enhancer 2 (MEF2), which positively regulates target gene transcription (reviewed in [22]). Although the role of MEF2 in CD34+ cells is unknown, MEF2 has been implicated as an important regulator of muscle, neuronal and immune cell function and differentiation [22]. We identified 11 members of the KRAB box transcription factors expressed at higher levels in the CD34+ cells from CAD patients and 4 apoptosis-related genes were expressed at lower levels. However, the number of genes was too low to reach statistical significance in a pathway level analysis.

Detailed analysis of the genes with a lower expression in CD34+ cells from CAD patients led us to conclude that signaling and migration through CXCR4 may be affected. SDF-1/CXCL12, the ligand for CXCR4, is the predominant chemotactic and retention factor for stem cells. The capacity of bone marrow-derived mononuclear cells to repair ischemic tissue by revascularization therefore critically depends on their expression of CXCR4 [23]. EPC from CAD patients were shown to have a reduced revascularization capacity, with diminished CXCR4 signaling, as Jak2 phosphorylation by CXCL12 is impaired, compared to EPC from healthy individuals, while the expression levels of CXCR4 are normal [24]. In this study, no difference in CXCR4 mRNA expression in CD34+ cells from CAD patients was observed. However, lower transcript levels of genes were found involved in signal transduction and effector functions of CXCR4, i.e. G proteins (several small GTPases), actin filament assembly, (including CDC42), and binding capacity (integrins and tetraspanins). CDC42 has been discovered as an important regulator of stem cell migration through CXCR4 signaling [25]. Therefore, the migratory capacity of CD34+ cells from CAD patients appears affected in signaling, as well as cytoskeleton rearrangements and adhesive properties.

The more activated phenotype and reduced migratory capacity of CD34+ in CAD patients may be explained by an *in vivo *activation by retinoic acid. All-trans retinoic acid (atRA), the active vitamin A metabolite, regulates the growth and differentiation of a variety of cell types and tissues including stem cells and neuronal tissues. RA is known to activate ERK5 (MAPK7), suggesting that the increased expression of ERK5 (MAPK7) in CD34+ cells from CAD patients may be related to an increased sensitivity to RA [26]. The transcript levels of the MAPK pathway correlated with the RA-induced genes, including RARα2, supportive for a causal relationship between RA and MAPK activation (Figure 4C, R = 0.66). Interestingly, during RA-induced neurogenesis, MAPkinase p38 activates MEF2 [27], providing a direct link between RA, MAPkinases, MEF2 activation, and differentiation.

The most significant upregulated RA-inducible gene in CD34+ cells from CAD patients was RAI1, a transcription factor which does not posses a DNA binding domain but likely influences differentiation through complex formation with other proteins mediating chromatin remodeling [28]. The increased expression of the RARα may contribute to RA responsiveness in CD34+ cells from CAD patients. In correspondence with an RA-induced profile in CD34+ cells from CAD patients, these cells showed a concomitant higher expression of the transcription factor GATA2, which has been reported to functionally cooperate with RARα in embryonic stem cells [29]. The relevance of its increased transcription in CD34+ cells in CAD patients is underscored by the association of a polymorphisms in GATA2 with familial early onset coronary artery disease [30]. Interestingly, administration of atRA in experimental nephritis diminishes migration of PBMC to the site of inflammation. It was further demonstrated that atRA inhibits cytoskeleton re-arrangements required for cell morphology changes, cell adhesion and migration [31]. These results, together with our observation that the genes involved in adhesion and migration strongly anti-correlated with RAI1, suggest that RA may have caused the low migratory profile in CD34+ cells from CAD patients. It is unclear where the CD34+ cells have received the activation signal from atRA. Measurements of atRA in mouse and humans samples indicated that concentrations are in the nanomolar range and are much higher in tissues (such as liver samples) than in serum [32,33]. Most likely atRA is present at biologically effective concentrations in the bone marrow, where CD34+ cells reside before they enter the bloodstream. CD34+ cells from bone marrow are responsive to atRA as their cytokine-dependent growth is inhibited, while differentiation is promoted by atRA [34]. The biological effects of atRA are highly dependent on the differentiation stage of the cell [35,36], which explains why only CD34+ cells show signs of atRA activation, and none of the more differentiated cell types.

### Limitations

Patients were carefully matched for several parameters, including medication and referall to a cardiologist. Because all individuals were suspected of having coronary artery disease, all were on aspirin and statin therapy. However, the controls were free of visible coronary artery disease at coronary angiography. Because the controls had the same symptoms as patients with severe 3-vessel CAD they may suffer from other underlying pathologies, which may have masked our analysis. However, a control group without symptoms would introduce a strong bias through a lack of matching for medication and risk factors for atherosclerosis.

Because of our careful matching, the number of patients and controls is limited. On the other hand, the paired analysis of CD34+ cells with other cells from the same individual strongly improves the power to detect differences.

## Conclusions

In general, hematopoietic progenitor cells may improve tissue repair in ischemic conditions by providing a micro-environmental niche allowing angiogenesis and neurogenesis from local more specific cell types [1-3]. The reduced adhesive/migratory capacity of circulating precursor cells in CAD patients may restrict their recruitment by CXCL12 to ischemic tissues, thereby limiting their role in tissue repair. The data are supportive for a causative role of retinoic acid in reducing the migratory capacity. A role for retinoic acid in atherosclerosis has been suggested by the identification of increased transcript levels of the retinoic acid receptor responder-1 gene (RARRES1) in unstable carotid endarterectomy plaque specimens, suggesting that cells within atherosclerotic plaques have been stimulated by retinoic acid [37]. Future research aimed at the functional consequences of retinoic acid stimulation of CD34+ subpopulations and isolated endothelial precursor cells could provide more insight in the affected migration and differentiation of precursor cells in CAD patients and ways to reverse this programming. Collectively, our data suggest that the capacities of precursor cells to support ischemic tissue repair might be improved by counteracting the RA signature, possibly by the development of an antagonist for the RARα2 receptor.

## Methods

### Patient selection

This study is a substudy of a previously published investigation [10]. It was approved by the institutional medical ethics committee and has been performed in accordance with the ethical standards laid down in the 1964 Declaration of Helsinki. All patients gave informed consent prior to their inclusion in the study. We selected 11 Caucasian patients without any sign of CAD on a coronary angiography. These patients are further referred to as "controls". Controls underwent angiography because of symptoms of chest pain and a high pretest-likelihood of atherosclerotic CAD, justifying the invasive diagnostic procedure. The controls were prospectively matched to 12 Caucasian patients with severe triple-vessel CAD on the basis of age, gender, race, smoking status, lipid-profile and medication ("atherosclerotic patients"). All patients and controls were on aspirin and statin treatment. Exclusion criteria were: myocardial infarction <4 weeks, diabetes mellitus, neoplastic disease and systemic inflammatory disease.

### Statistical analysis

Fisher's exact test was used for testing association in 2 × 2 contingency tables. For single genes, comparisons among the two groups were performed by Student's t-test and when variances were different, a Welch's correction was applied.

### Isolation, culture and gene expression analysis of CD34+ cells

From all 23 patients and controls, 60 ml peripheral blood was drawn from the arterial sheath after the angiography but before possible coronary intervention. Using immunomagnetic beads (Dynabeads, Invitrogen, Carlsbad, CA), CD34+ precursor cells, CD14+ monocytes, and CD4+ T cells were positively isolated for direct cell lysis, while negatively isolated monocytes were taken into culture towards macrophages for 20 h. mRNA from all cell populations was amplified and biotinylated using the Illumina TotalPrep RNA amplification Kit (Ambion, Austin, TX). Six technical replicates were performed. From CD34+ cells we obtained sufficient amounts of mRNA for one hybridization only. Samples were randomly hybridized to Sentrix HumanRef-8 Expression bead chip arrays (Illumina, San Diego, CA), followed by scanning and feature extraction, performed at ServiceXS (Leiden, The Netherlands), as previously described [10].

### Analysis of gene array data

Normalization and statistical analysis of the gene array bead summary data was carried out with established methods using the limma package [38] and in-house scripts in R/Bioconductor [39]. Bead summary intensities were RMA-background corrected [40], log2-transformed and normalized using quantile normalization [41]. For the identification of over- or under-expressed genes in CD34+ cells, we made three separate pair-wise comparisons between CD34+ cells and one of the other three cell types (CD4+, CD14+, or CD14+-differentiated macrophages) from the same individual (n = 23) in a two-class paired analysis using Significance Analysis of Microarrays (SAM) [11]. Because genes may be reliably expressed in only one of the two mononuclear cell types used in the comparisons, we included only genes in the analysis if the average expression in at least one of the two cell types was > 6 (Log2). Genes with a false discovery rate (FDR) < 5% were considered significantly different. Genes that consistently showed differential over- or under expression between CD34+ versus all other cell types were selected as CD34+ -enriched genes. For the comparison of the CD34+ gene expression profiles between CAD patients and controls we included genes which showed an average log2 expression > 6, in a two-class unpaired analysis in SAM.

Hierarchical clustering [42] of samples was used to visualize the correlation of co-expressed genes in Treeview (available at http://rana.lbl.gov/EisenSoftware.htm).

For an interpretation of the biological processes that are represented by the genes that show a significantly different level of expression in CD34+ cells, compared to the other cell types, and for genes that differed in expression between CAD patients and controls, we used Gene Ontology analysis using the PANTHER (**P**rotein **AN**alysis **TH**rough **E**volutionary **R**elationships) Classification System at http://www.pantherdb.org/[43]. PANTHER uses the binomial statistics tool to compare our gene list to a reference list (NCBI: Homo sapiens genes) to determine the statistically significant over-representation of functional groups of genes. A Bonferroni correction was applied to adjust for multiple testing.

P values (Bonferroni-corrected) < 0.05 were considered significant

Pathway analysis was performed by the Metacore™ mapping tools, developed by GeneGo (GeneGO, St Joseph, MI, http://www.genego.com/). Data mining in Metacore™ is based on a manually curated database of human protein-protein, protein-DNA interactions, transcription factors, signaling pathways and metabolic pathways. Calculation of statistical significance are based on p-values which are defined as the probability of a given number of genes from the input list to match a certain number of genes in the maps. All microarray data have been submitted to the Gene Expression Omnibus (GEO) under accession number GSE9820.

## Authors' contributions

TvdPK performed the data analysis and drafted the manuscript. SHS participated in patient recruitment, cell isolation, acquisition and interpretation of data, and was involved in drafting the manuscript. JOF participated in cell isolation and acquisition and interpretation of data. PDM was involved in the data analysis. JMB, AMvdL and TAL participated in acquisition and interpretation of data. JJP and NvR participated in the design and coordination of the study, patient selection and recruitment and drafting the manuscript. AJGH participated in the design and coordination of the project, was involved in data analysis and interpretation and drafting of the manuscript. All authors read and approved the final manuscript.

## Supplementary Material

Additional file 1 **Supplemental figure S1**. Hierarchical cluster analysis clearly separates the five circulating cell populations. Unsupervised hierarchical clustering of all genes expressed in the individual circulating cell types. Genes are represented in rows, cell types in columns. Red indicates a relative high expression, green a low expression level. Click here for file

Additional file 2 **Supplemental table S1**. A list of the genes which are expressed at significant lower levels in CD34+ cells compared to more differentiated cells, enriched in the apoptosis signaling pathway. Click here for file

Additional file 3 **Supplemental figure S2**. CD34+ cells from CAD patients display an impaired integrin-mediated cell adhesion and migration pathway. Genes with a significant lower expression in CD34+ cells from CAD patients compared to controls are significantly enriched in the Integrin-mediated cell adhesion and migration pathway (P = 6.1 × 10^-10^) and indicated by a red arrow and encircled for clarity. Click here for file

Additional file 4 **Supplemental figure S3**. CD34+ cells from CAD patients display an affected cytoskeleton remodeling pathway. Genes with a lower expression in CD34+ cells from CAD patients compared to controls are significantly enriched in the Cytoskeleton remodeling pathway (p = 1.6 × 10^-4^). The encircled genes with red arrows indicate the genes with a significantly lower expression in CD34+ cells from CAD patients. Click here for file
