# Supplementary figures and images for: Expression of a retinoic acid signature in circulating CD34 cells from coronary artery disease patients
Source: BMC Genomics. 2010 Jun 21;11:388. doi: 10.1186/1471-2164-11-388 (PMC2901320; doi:10.1186/1471-2164-11-388)

## Slide 1
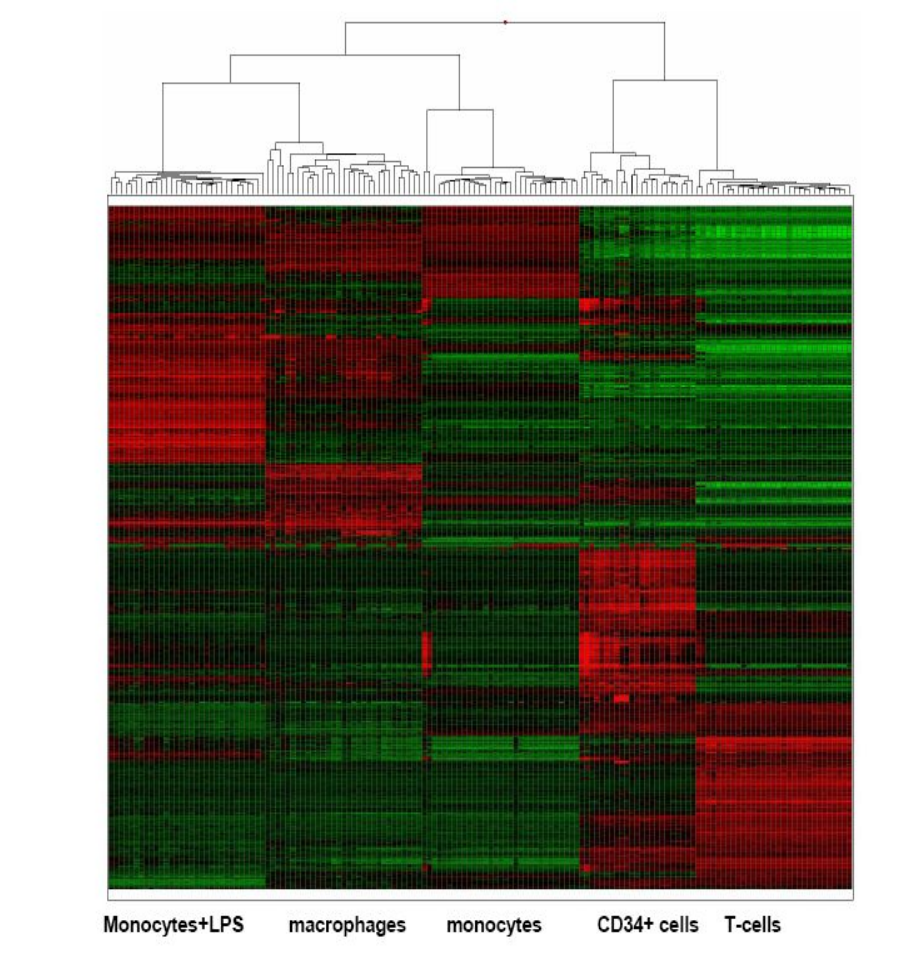

Supplement: Additional file 1 — Supplemental figure S1. Hierarchical cluster analysis clearly separates the five circulating cell populations. Unsupervised hierarchical clustering of all genes expressed in the individual circulating cell types. Genes are represented in rows, cell types in columns. Red indicates a relative high expression, green a low expression level. [file 1471-2164-11-388-S1.PPT]

## Slide 1
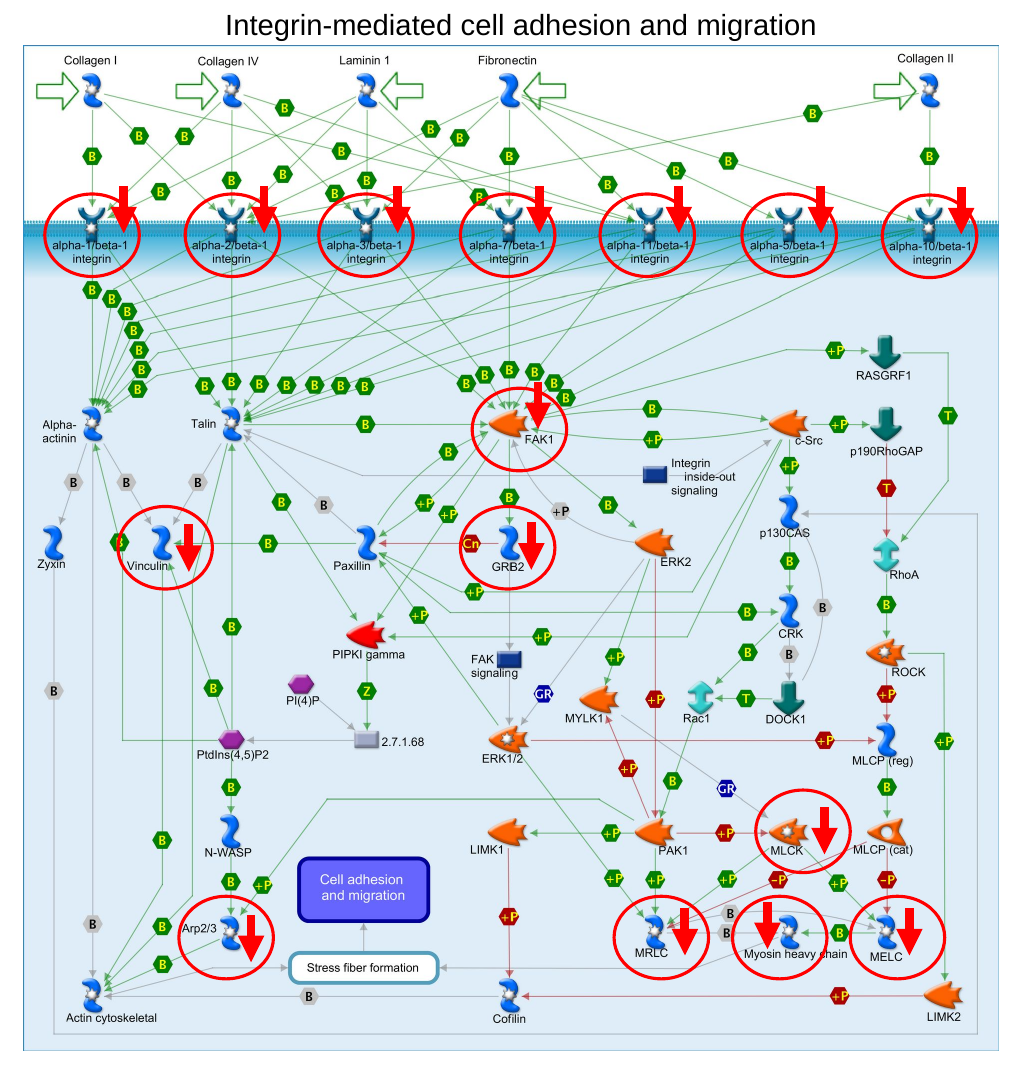

Integrin-mediated cell adhesion and migration

Supplement: Additional file 3 — Supplemental figure S2. CD34+ cells from CAD patients display an impaired integrin-mediated cell adhesion and migration pathway. Genes with a significant lower expression in CD34+ cells from CAD patients compared to controls are significantly enriched in the Integrin-mediated cell adhesion and migration pathway (P = 6.1 × 10^-10^) and indicated by a red arrow and encircled for clarity. [file 1471-2164-11-388-S3.PPT]

## Slide 1
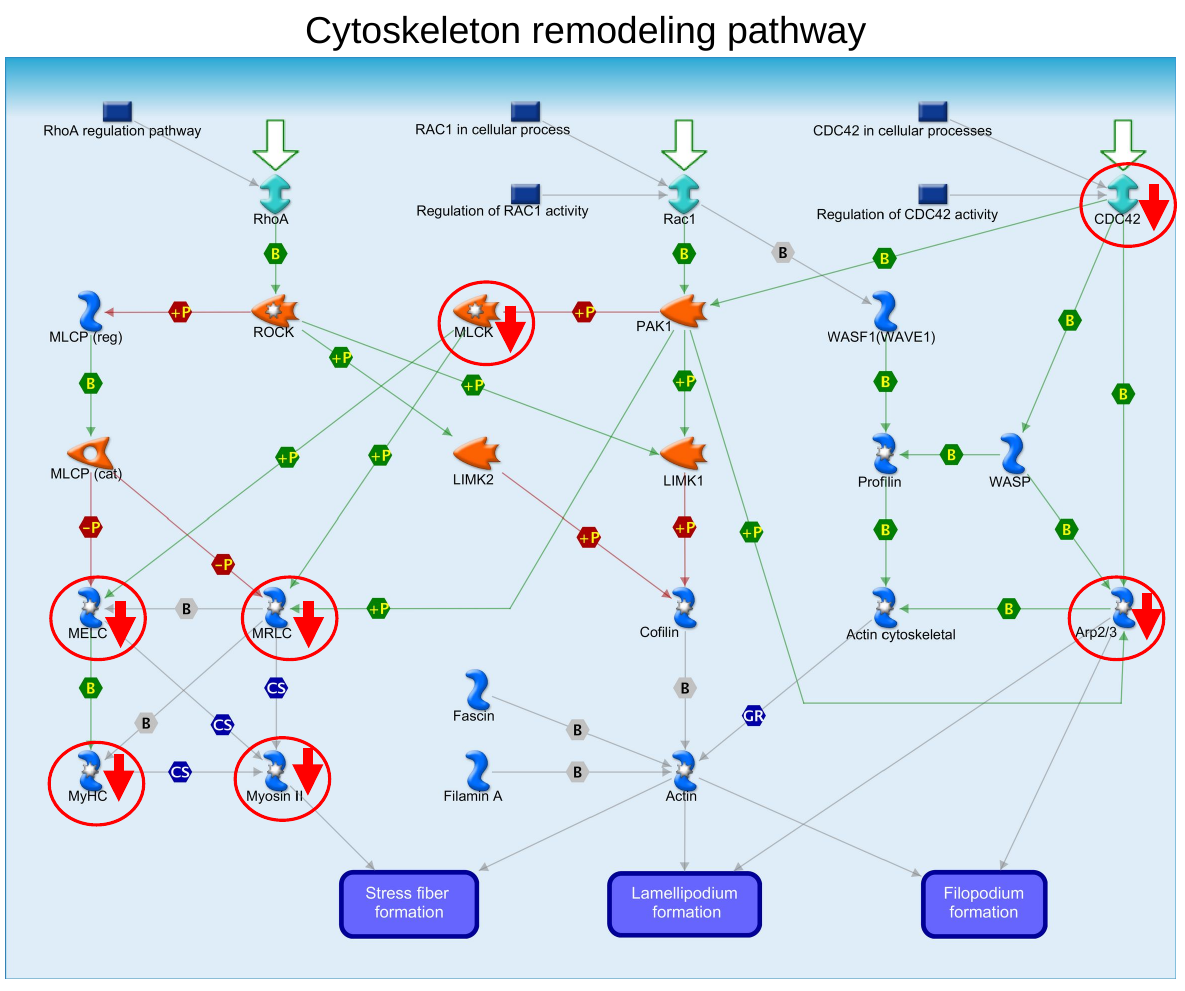

Cytoskeleton remodeling pathway

Supplement: Additional file 4 — Supplemental figure S3. CD34+ cells from CAD patients display an affected cytoskeleton remodeling pathway. Genes with a lower expression in CD34+ cells from CAD patients compared to controls are significantly enriched in the Cytoskeleton remodeling pathway (p = 1.6 × 10^-4^). The encircled genes with red arrows indicate the genes with a significantly lower expression in CD34+ cells from CAD patients. [file 1471-2164-11-388-S4.PPT]
